# The Yeast Sks1p Kinase Signaling Network Regulates Pseudohyphal Growth and Glucose Response

**DOI:** 10.1371/journal.pgen.1004183

**Published:** 2014-03-06

**Authors:** Cole Johnson, Hye Kyong Kweon, Daniel Sheidy, Christian A. Shively, Dattatreya Mellacheruvu, Alexey I. Nesvizhskii, Philip C. Andrews, Anuj Kumar

**Affiliations:** 1 Department of Molecular, Cellular, and Developmental Biology, University of Michigan, Ann Arbor, Michigan, United States of America; 2 Department of Biological Chemistry, University of Michigan Medical School, Ann Arbor, Michigan, United States of America; 3 Department of Pathology, University of Michigan Medical School, Ann Arbor, Michigan, United States of America; 4 Department of Computational Medicine and Bioinformatics, University of Michigan Medical School, Ann Arbor, Michigan, United States of America; 5 Department of Chemistry, University of Michigan, Ann Arbor, Michigan, United States of America; Syracuse, United States of America

## Abstract

The yeast *Saccharomyces cerevisiae* undergoes a dramatic growth transition from its unicellular form to a filamentous state, marked by the formation of pseudohyphal filaments of elongated and connected cells. Yeast pseudohyphal growth is regulated by signaling pathways responsive to reductions in the availability of nitrogen and glucose, but the molecular link between pseudohyphal filamentation and glucose signaling is not fully understood. Here, we identify the glucose-responsive Sks1p kinase as a signaling protein required for pseudohyphal growth induced by nitrogen limitation and coupled nitrogen/glucose limitation. To identify the Sks1p signaling network, we applied mass spectrometry-based quantitative phosphoproteomics, profiling over 900 phosphosites for phosphorylation changes dependent upon Sks1p kinase activity. From this analysis, we report a set of novel phosphorylation sites and highlight Sks1p-dependent phosphorylation in Bud6p, Itr1p, Lrg1p, Npr3p, and Pda1p. In particular, we analyzed the Y309 and S313 phosphosites in the pyruvate dehydrogenase subunit Pda1p; these residues are required for pseudohyphal growth, and Y309A mutants exhibit phenotypes indicative of impaired aerobic respiration and decreased mitochondrial number. Epistasis studies place *SKS1* downstream of the G-protein coupled receptor *GPR1* and the G-protein *RAS2* but upstream of or at the level of cAMP-dependent PKA. The pseudohyphal growth and glucose signaling transcription factors Flo8p, Mss11p, and Rgt1p are required to achieve wild-type *SKS1* transcript levels. *SKS1* is conserved, and deletion of the *SKS1* ortholog *SHA3* in the pathogenic fungus *Candida albicans* results in abnormal colony morphology. Collectively, these results identify Sks1p as an important regulator of filamentation and glucose signaling, with additional relevance towards understanding stress-responsive signaling in *C. albicans*.

## Author Summary

Eukaryotic cells respond to nutritional and environmental stress through complex regulatory programs controlling cell metabolism, growth, and morphology. In the budding yeast *Saccharomyces cerevisiae*, conditions of limited nitrogen and/or glucose can initiate a dramatic growth transition wherein the yeast cells form extended multicellular filaments resembling the true hyphal tubes of filamentous fungi. The formation of these pseudohyphal filaments is governed by core regulatory pathways that have been studied for decades; however, the mechanism by which these signaling systems are integrated is less well understood. We find that the protein kinase Sks1p contributes to the integration of signals for nitrogen and/or glucose limitation, resulting in pseudohyphal growth. We implemented a mass spectrometry-based approach to profile phosphorylation events across the proteome dependent upon Sks1p kinase activity and identified phosphorylation sites important for mitochondrial function and pseudohyphal growth. Our studies place Sks1p in the regulatory context of a well-known pseudohyphal growth signaling pathway. We further find that *SKS1* is conserved and required for stress-responsive colony morphology in the principal opportunistic human fungal pathogen *Candida albicans*. Thus, Sks1p is part of the mechanism integrating glucose-responsive cell signaling and pseudohyphal growth, and its function is required for colony morphology linked with virulence in *C. albicans*.

## Introduction

Multiple fungal species exhibit complex morphological changes in response to environmental conditions, generating multicellular forms or structures critical to the respective life cycles of these organisms [1]–[3]. These morphological transitions have been linked to virulence in several human and plant fungal pathogens, including *Candida albicans*, *Cryptococcus neoformans*, *Aspergillus fumigates*, and *Ustilago maydis* [4]–[6]. In particular, several lines of study have established that the formation of hyphal filaments is required for virulence in the opportunistic human fungal pathogen *C. albicans* [7]–[10]. The budding yeast *Saccharomyces cerevisiae* also exhibits a morphogenetic transition from its typical form to a filamentous state [11], and the study of this dimorphism in *S. cerevisiae* has contributed considerably to our understanding of important cell signaling mechanisms, while also providing insight into the molecular basis of fungal pathogenicity [3].

The morphological transition in *S. cerevisiae* is pronounced: yeast cells undergoing pseudohyphal growth are elongated and remain connected after cytokinesis, forming multicellular chains, or filaments [11]–[15]. These filaments of connected cells can spread out along the surface of a solid growth substrate as well as invade the substrate [11] and are referred to as pseudohyphae, since they resemble the hyphae of other fungal species but lack the structure of a true hyphal tube [16]. Strains of *S. cerevisiae* competent to undergo pseudohyphal growth (e.g., the Σ1278b strain used here) initiate this transition in response to conditions of nitrogen limitation and/or glucose limitation [11], [17], [18]. Consequently, pseudohyphal growth is considered to be an adaptive mechanism, enabling non-motile yeast cells to forage for nutrients when local resources become limited [19]. The morphological changes associated with pseudohyphal growth are driven by a host of altered developmental processes, including a delay in the G2/M cell-cycle transition that produces the elongated cell morphology [20]–[22], a switch to a unipolar budding pattern [11], [20], and increased cell-cell adhesion [11].

At least 700 single gene deletions in the filamentous Σ1278b strain of *S. cerevisiae* result in pseudohyphal growth phenotypes [23], [24], and classic studies have established three well-studied signaling pathways as regulators of pseudohyphal differentiation: the mitogen-activated protein kinase (MAPK) pathway, the cAMP-dependent protein kinase A (PKA) pathway, and the sucrose non-fermentable (SNF) pathway. The yeast pseudohyphal growth MAPK pathway consists of the MAPKKK Ste11p, the MAPKK Ste7p, and the MAPK Kss1p [12], [13], [25]–[27]. Ste11p is phosphorylated by the p21-activated kinase Ste20p [28], and Kss1p phosphorylates the key heterodimeric transcription factor Ste12p/Tec1p [29], [30]. In *S. cerevisiae*, PKA consists of the regulatory subunit Bcy1p and one of three catalytic subunits, Tpk1p, Tpk2p, and Tpk3p [31], [32]. Deletion of *TPK2* results in a loss of pseudohyphal growth, and Tpk2p has been implicated most extensively in filamentation and the response to nitrogen stress [31]. Tpk2p phosphorylates the transcription factor Flo8p, which is required for pseudohyphal growth [32], [33]. Snf1p is a member of the AMP-activated kinase family and regulates transcriptional changes associated with glucose derepression [34], [35]. Snf1p regulates the key pseudohyphal growth effector *FLO11* through repression of the negative regulators Nrg1p and Nrg2p [35]. The Kss1p MAPK pathway and PKA also activate *FLO11* transcription through Ste12p/Tec1p and Flo8p, respectively [36]–[38].

Notably, each pathway above is involved in the cellular response to nutrient availability [18], [39], [40]. In particular, glucose, the preferred carbon source of budding yeast, effects changes in transcription principally through the Ras/PKA pathway [41], and glucose limitation activates the heterotrimeric Snf1p kinase complex through phosphorylation of T210 in Snf1p [42], [43]. The mechanisms by which these signals are then propagated and executed to elicit pseudohyphal differentiation, however, are still under investigation. Studies from Bisson and colleagues [44] identified the *SKS1* gene, encoding a Ser/Thr kinase, as a multicopy suppressor of *snf3*Δ; mutants deleted for *SNF3* are defective in high-affinity glucose transport and cannot grow by fermentation on low-glucose medium. Yang and Bisson also demonstrated that Sks1p kinase activity was required for phenotypic suppression of *snf3*Δ. Recent work in our lab indicated that Sks1p undergoes a localization shift to the nucleus during butanol-induced pseudohyphal growth and that its kinase activity is required for wild-type localization of Ksp1p, a stress granule-associated protein with pseudohyphal growth deletion phenotypes [45]. Thus, *SKS1* may regulate both glucose-responsive signaling and pseudohyphal development, with the potential to serve as an integrator between these interrelated signaling processes.

## Results

### Sks1p kinase activity is required for pseudohyphal growth

**Figure 1:**
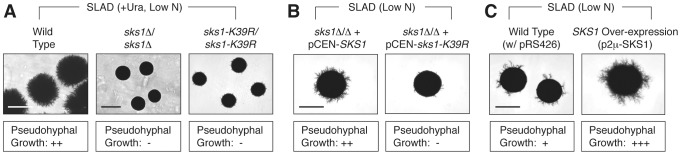
Phenotypic analysis of *SKS1* mutants. A) Homozygous diploid *sks1*Δ/Δ deletion mutants and *sks1-K39R*/*sks1-K39R* kinase-dead mutants exhibit a loss of surface-spread filamentation relative to a wild-type strain under identical growth conditions of nitrogen limitation (SLAD); uracil was added to complement an auxotrophy in the background strain. B) Addition of a centromeric plasmid bearing wild-type *SKS1* to the *sks1*Δ/Δ mutant restores pseudohyphal growth, while addition of the same plasmid bearing the kinase-dead *sks1-K39R* allele fails to restore surface-spread filamentation. C) Overexpression of *SKS1* with its native promoter from a high-copy 2μ plasmid induces exaggerated surface-spread filamentation in a wild-type strain on nitrogen-limiting SLAD medium. The degree of observed pseudohyphal filamentation is indicated below each image, with the “−” indicating an absence of filamentation and “+++” indicating the strongest observed pseudohyphal growth. Scale bar, 2 mm.

We initially assessed the role of Sks1p in pseudohyphal growth through a series of phenotypic studies analyzing pseudohyphal filamentation in *SKS1* mutants under conditions of nitrogen limitation. For this work, we constructed homozygous diploid *sks1*Δ/Δ, and *sks1*-*K39R*/*sks1*-*K39R* mutants in the filamentous Σ1278b genetic background, with the latter strain containing a site-directed mutation yielding greatly diminished kinase activity. On low-nitrogen (SLAD) media, loss of either the gene or its kinase activity resulted in decreased surface-spread filamentation relative to an isogenic wild-type strain (Figure 1A). The introduction of a centromeric plasmid bearing wild-type *SKS1* under transcriptional control of its native promoter was able to rescue the loss of pseudohyphal growth exhibited by the *sks1*Δ/Δ mutant, while introduction of a similar plasmid bearing the kinase-dead variant of *SKS1* (*sks1-K39R*) was unable to restore wild-type filamentation (Figure 1B). Overexpression of *SKS1* from a high-copy 2μ plasmid induced hyper-filamentation under conditions of nitrogen limitation (Figure 1C).

### Identification of the Sks1p signaling network

**Figure 2:**
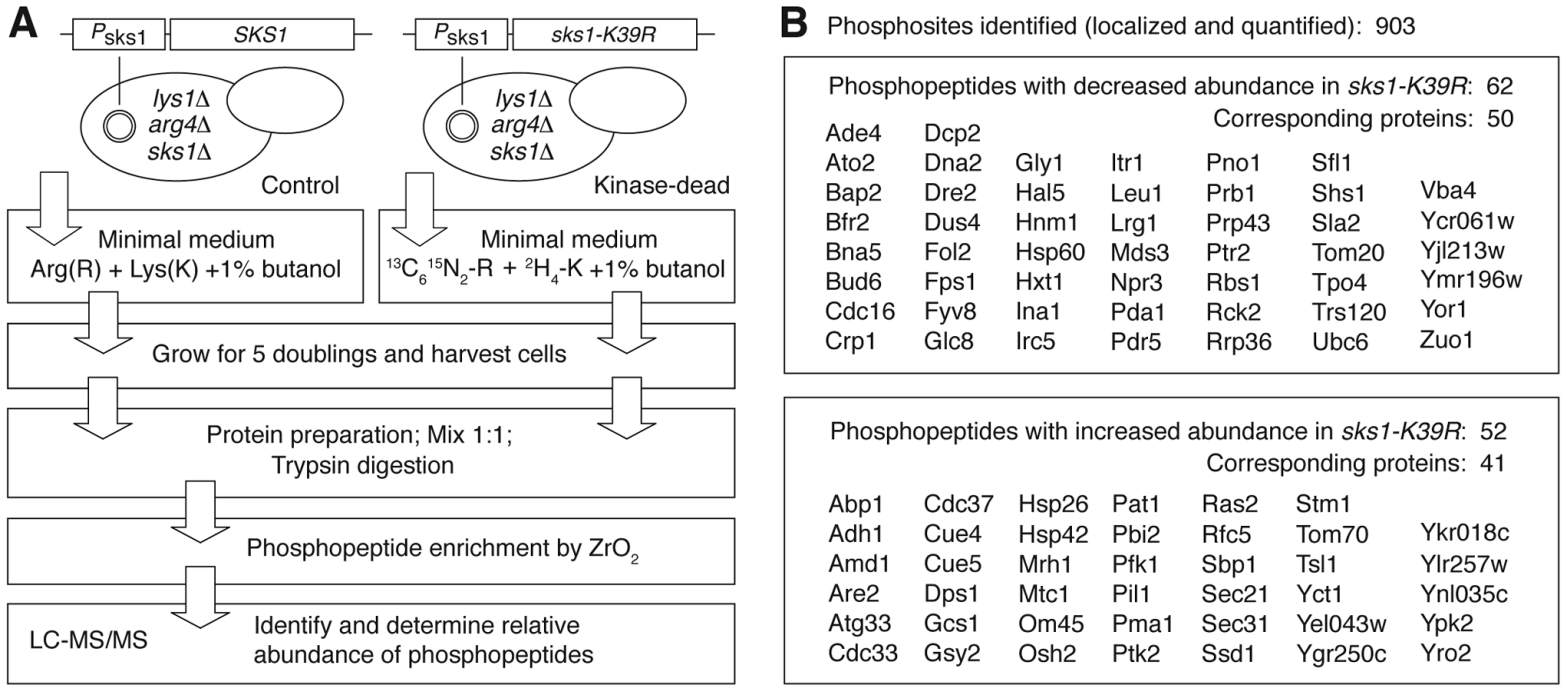
Quantitative phosphoproteomic analysis of the Sks1p signaling network in the filamentous Σ1278b genetic background. A) Schematic overview of major steps in the SILAC-based mass spectrometric analysis of Sks1p signaling; strain auxotrophies are indicated in the control and kinase-dead strains. B) A listing of proteins exhibiting decreased or increased phosphopeptide abundance after enrichment from a strain carrying the *sks1-K39R* kinase-dead allele relative to a strain carrying wild-type *SKS1*.

To determine the molecular basis of Sks1p kinase regulation of peudohyphal growth, we analyzed the Sks1p kinase signaling network by quantitative phosphoproteomics. Our approach was straightforward; we implemented a mass spectrometry-based method utilizing stable isotope labeling of amino acids in cell culture (SILAC) to identify proteins differentially phosphorylated upon loss of Sks1p kinase activity [46], [47]. As outlined in Figure 2A, a strain that was wild-type with respect to *SKS1* and an otherwise isogenic strain carrying the *sks1-K39R* allele in the filamentous Σ1278b background were made auxotrophic for arginine and lysine; the wild-type and *sks1*kinase-dead strains were subsequently grown in triplicate for five cell doublings in media containing unlabeled or labeled arginine and lysine, respectively, in the presence of butanol to induce pseudohyphal growth. Prepared proteins from both sets of samples were enriched for phosphopeptides, and differences in phosphopeptide abundance between the wild-type and kinase-dead samples were determined by mass spectrometry.

By this approach, we profiled 903 phosphorylation sites across the yeast proteome, identifying 114 phosphopeptides differentially abundant upon enrichment from the *sks1* kinase-dead strain relative to wild-type (Figure 2B). These peptides correspond to 91 proteins in total, encompassing phosphorylation events directly and indirectly dependent upon the presence of Sks1p kinase activity. Interestingly, by comparing the phosphorylation sites determined in this study against *S. cerevisiae* phosphorylation sites reported in public databases, we identified 39 new phosphorylation sites in the yeast proteome. Table 1 presents novel phosphorylation sites in peptides differentially abundant upon phosphopeptide enrichment from the *sks1-K39R* mutant relative to wild type. A listing of phosphopeptides is presented in Dataset S1, and the full mass spectrometry dataset can be accessed at ProteomeXchange (dataset identifier PXD000414).

**Table 1:** Previously unreported phosphosites from peptides differentially abundant in *sks1-K39R*.

Protein	Phosphosite	Localization Probability^a^	Abundance *sks1-K39R* b
Bul2	ASDSQDDDIRSASTT(ph)NLDR	0.78	0.50
Cdc16	NS(ph)MFGSTIPST(ph)LRKVSLQR	0.95	0.091
Dna2	HQLQEVFGQAQS(ph)R	1.0	0.14
Dse4	VS(ph)AASHSPLSVSPK	0.96	1.5
Est2	LFNVVNAS(ph)R	1.0	0.52
Irc5	DNSNSDDEEHS(ph)SKKR	0.99	0.15
Lsm4	RPYS(ph)QNR	0.99	1.5
Mds3	NS(ph)S(ph)KAVRQEGR	1.0	0.27
Nth2	ALPQLEMLGGLVACT(ph)EKSR	0.97	0.33
Nup157	MYS(ph)TPLKKR	0.99	0.30
Pal1	RGGDT(ph)QDAIK	1.0	0.31
Pat1	DLS(ph)PEEQR	1.0	2.0
Ras2	QAINVEEAFY(ph)T(ph)LARLVR	1.0	5.8
Rfc5	NIRLIMVCDS(ph)MSPIIAPIK	0.99	8.1
Sas10	S(ph)VRAVY(ph)S(ph)GGQS(ph)GVYEGEKTGIK	0.99	0.51
Ygr250c	RGNLSSSDDDDQSQT(ph)DNSSK	0.77	1.8
Ylr177w	RNTQPVLNLHPAAAPT(ph)NDAGLAVVDGK	0.87	0.43

a Phosphosites indicating a localization probability of *p*>0.75.

b Phosphopeptide abundance as *sks1-K39R*/WT ratio.

### Sks1p signaling network connectivity

**Figure 3:**
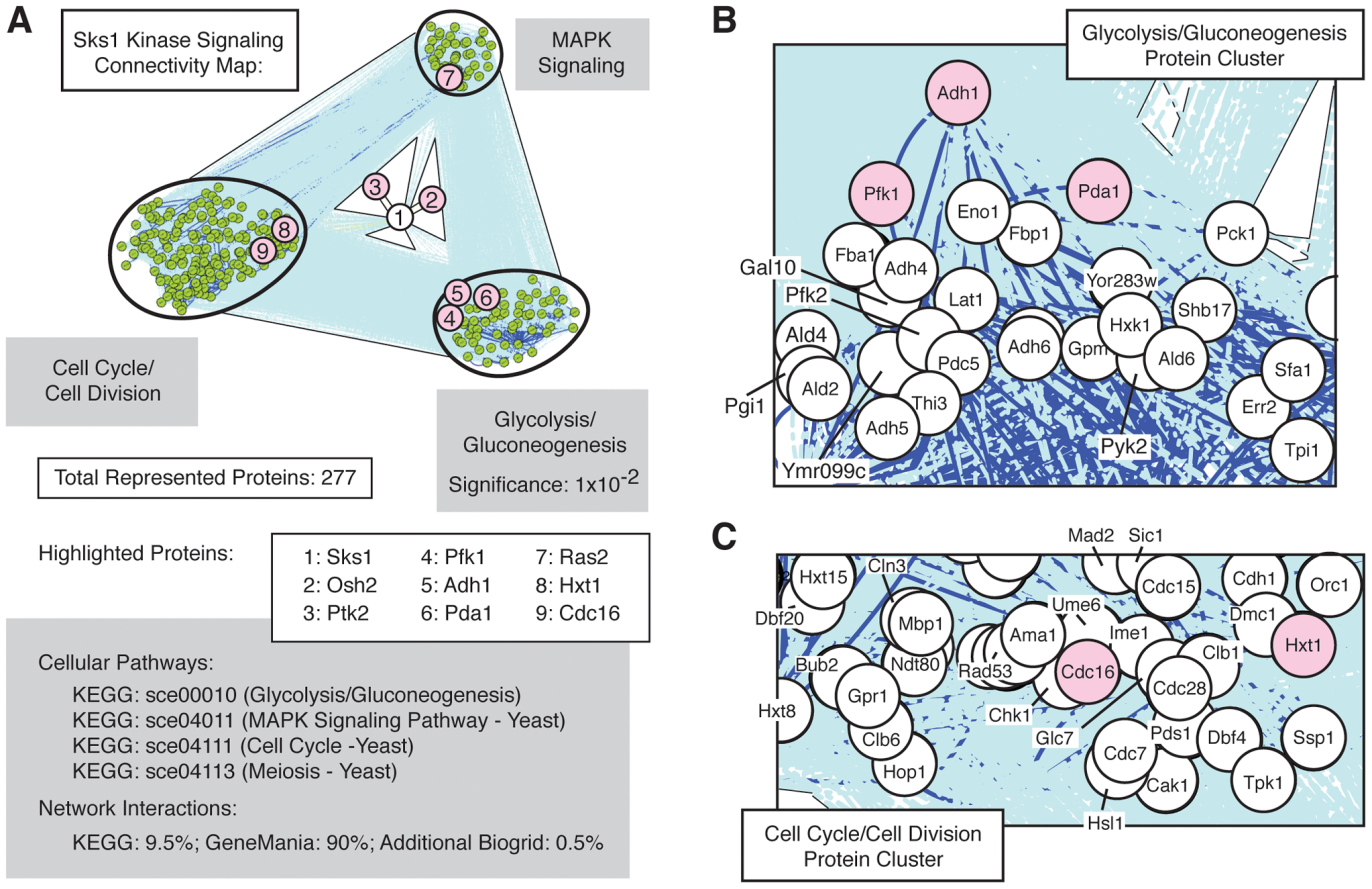
Network connectivity of Sks1p kinase signaling. A) Network connectivity map constructed by expanding connections involving Sks1p-dependent phosphoproteins through genetic and physical interactions from KEGG, BioGrid, and GeneMania. Clusters of proteins enriched for the indicated KEGG signaling pathways are shown. Numbered proteins identify Sks1p and a subset of proteins exhibiting Sks1p-dependent phosphorylation by mass spectrometry (shaded red) within the context of the larger connected network. As indicated, the KEGG glycolysis/gluconeogenesis pathway was enriched in the subset of Sks1p-dependent phosphoproteins identified by mass spectrometry. Proteins within the network clustered around B) the KEGG glycolysis/gluconeogenesis pathway and C) the KEGG cell cycle and meiosis pathways are highlighted.

The set of Sks1p-dependent phosphoproteins identified in this study is statistically enriched (*p*-value of 10^−2^) for the cellular pathway enabling glycolysis and gluconeogenesis (Figure 3A), as defined in the KEGG database; the glycolysis and gluconeogenesis pathway is annotated in KEGG as sce00010. To gain a better understanding of the means through which Sks1p-dependent glucose signaling impacts additional cell processes and pathways, we used the glycolysis/gluconeogenesis pathway as a starting point for the construction of an interaction network. In brief, reported genetic and physical interactions with components of the KEGG glycolysis/gluconeogenesis pathway were incorporated and expanded until Sks1p was included in the network as well as MAPK signaling and cell cycle pathways known to be required for wild-type pseudohyphal growth (Figure 3B and C). The resulting interaction network structure indicates two points. First, the clusters of genes enriched in MAPK signaling and cell cycle control exhibit a greater number of genetic and physical interactions between each other than with the cluster of genes enriched for glycolysis/gluconeogenesis; this is evident visually from the dark blue lines in Figure 3A indicating increased interaction density connecting the MAPK- and cell cycle-enriched clusters. Second, the network distance of Sks1p to proteins exhibiting Sks1p-dependent phosphorylation in the glycolysis/gluconeogenesis-enriched cluster is typically small and establishes a stronger interconnection between Sks1p and this cluster than with clusters enriched for MAPK signaling and cell cycle regulation. This result is consistent with the observed enrichment for the KEGG glycolysis/gluconeogenesis pathway in the set of proteins exhibiting Sks1p-dependent phosphorylation. The interactions used to construct this network are presented in Dataset S2.

### Phenotypic analysis of Sks1p-dependent phosphorylation sites

**Figure 4:**
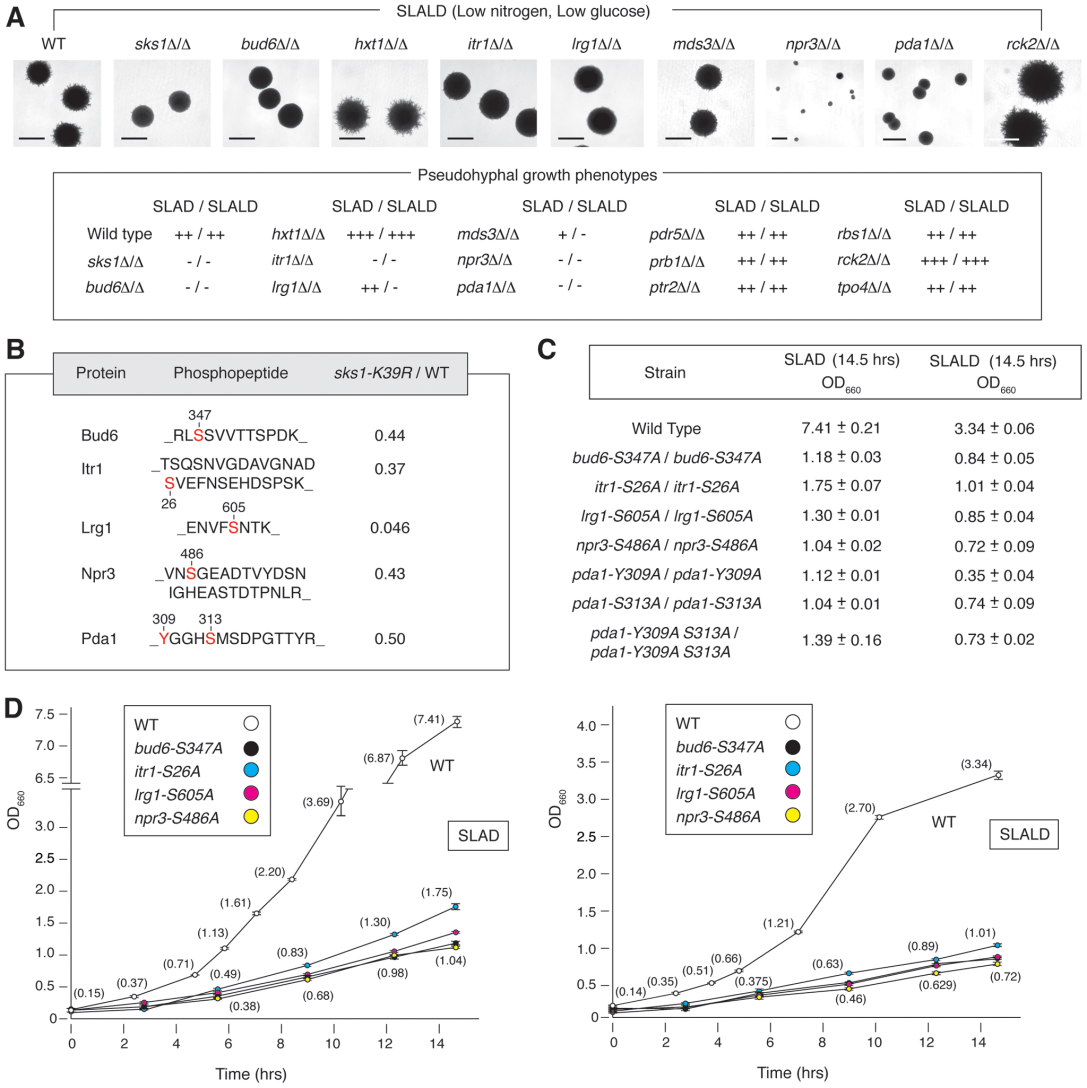
Phenotypic analysis of homozygous diploid mutants corresponding to a subset of Sks1p-dependent phosphoproteins identified by mass spectrometry. A) The indicated homozygous diploid deletion mutants exhibit surface-spread pseudohyphal filamentation phenotypes on SLAD and SLALD medium relative to a wild-type strain. The degree of observed pseudohyphal growth is indicated, with the “−” corresponding to an absence of filamentation and “+++” indicating the strongest observed pseudohyphal growth. Scale bars representing 2 mm are indicated. The *pda1*Δ/Δ strain formed slightly smaller colonies than wild type, and *npr3*Δ/Δ mutants formed markedly small colonies. B) Amino acid sequences identifying Sks1p-dependent phosphorylation sites selected for further phenotypic analysis. The ratio indicates abundance upon phosphopeptide enrichment from the *sks1-K39R* mutant relative to wild-type. All homozygous diploid integrated point mutants demonstrated a significant fitness defect compared to wild-type when grown in liquid media under nitrogen-limiting (SLAD) or nitrogen- and glucose-limiting (SLALD) conditions as indicated by C) the long-term cell titer and D) growth curves for each condition. Full values for the growth curves shown here are provided in Supplementary Tables S3 through S6.

As a first step towards identifying the phosphorylation events responsible for the filamentation defect observed in *sks1-K39R*, we screened a panel of yeast proteins exhibiting Sks1p-dependent phosphorylation for phenotypes similar to those of genes whose deletion affects pseudohyphal growth. Genes were selected by cross-referencing the list of phosphoproteins identified by our mass spectrometry study with genes identified as having pseudohyphal growth phenotypes [23], [24]. For this analysis, we prioritized genes with a role in glucose signaling. Homozygous diploid gene deletions were generated and screened for surface-spread filamentation under conditions of nitrogen limitation (SLAD medium) and nitrogen/glucose limitation (SLALD medium). Wild-type *S. cerevisiae* of the Σ1278b genetic background exhibits surface-spread filamentation on both SLAD and SLALD medium, and the homozygous *sks1*Δ/Δ mutant displays a loss of filamentation on both media. Figure 4A indicates deletion mutants exhibiting pseudohyphal growth phenotypes under these conditions. Strains deleted for *BUD6*, *ITR1*, *MDS3*, *NPR3*, and *PDA1* displayed significantly decreased pseudohyphal growth on both SLAD and SLALD medium; *lrg1*Δ/Δ mutants exhibited decreased pseudohyphal growth under conditions of nitrogen/glucose limitation. These genes contribute to pathways and cell processes required for pseudohyphal growth. *MDS3* and *NPR3* are TOR pathway components [48], [49], and *LRG1* encodes a putative GTPase-activating protein involved in the Pkc1p signaling pathway controlling cell wall integrity [50]. Bud6p is a polarity protein required for budding [51], and Itr1p is a myo-inositol transporter [52]; Pda1p is a subunit of the pyruvate dehydrogenase complex and will be discussed below. As indicated in Figure 4A, deletion of the *HXT1* gene encoding a low-affinity glucose transporter yielded hyperactive pseudohyphal growth on SLAD and SLALD media. Deletion of *RCK2* encoding a kinase responsive to oxidative and osmotic stress resulted in increased pseudohyphal growth; a similar phenotype has been observed upon decreased activity of the osmo-responsive Hog1p MAPK pathway [53].

To assess the functional significance of Sks1p-dependent phosphorylation sites, we constructed homozygous diploid strains containing chromosomal point mutations in *BUD6*, *ITR1*, *LRG1*, *NPR3*, and *PDA1*, substituting a non-phosphorylatable residue for the Sks1p-dependent phosphorylation site (Figure 4B). Corresponding phosphopeptides for each phosphorylation site exhibited decreased abundance upon zirconium dioxide enrichment in the kinase-dead *sks1-K39R* mutant. These integrated point mutants were assayed for fitness in SLAD and SLALD media, and each mutant exhibited a fitness defect relative to wild-type (Figure 4C and D), indicating that the mutated residues are necessary for optimal response to nitrogen and nitrogen/glucose limitation. Growth and growth rates for these strains in synthetic complete (SC) media are provided in Tables S5 and S6.

### Pda1p residues Y309 and S313 are necessary for pseudohyphal differentiation and respiratory growth

**Figure 5:**
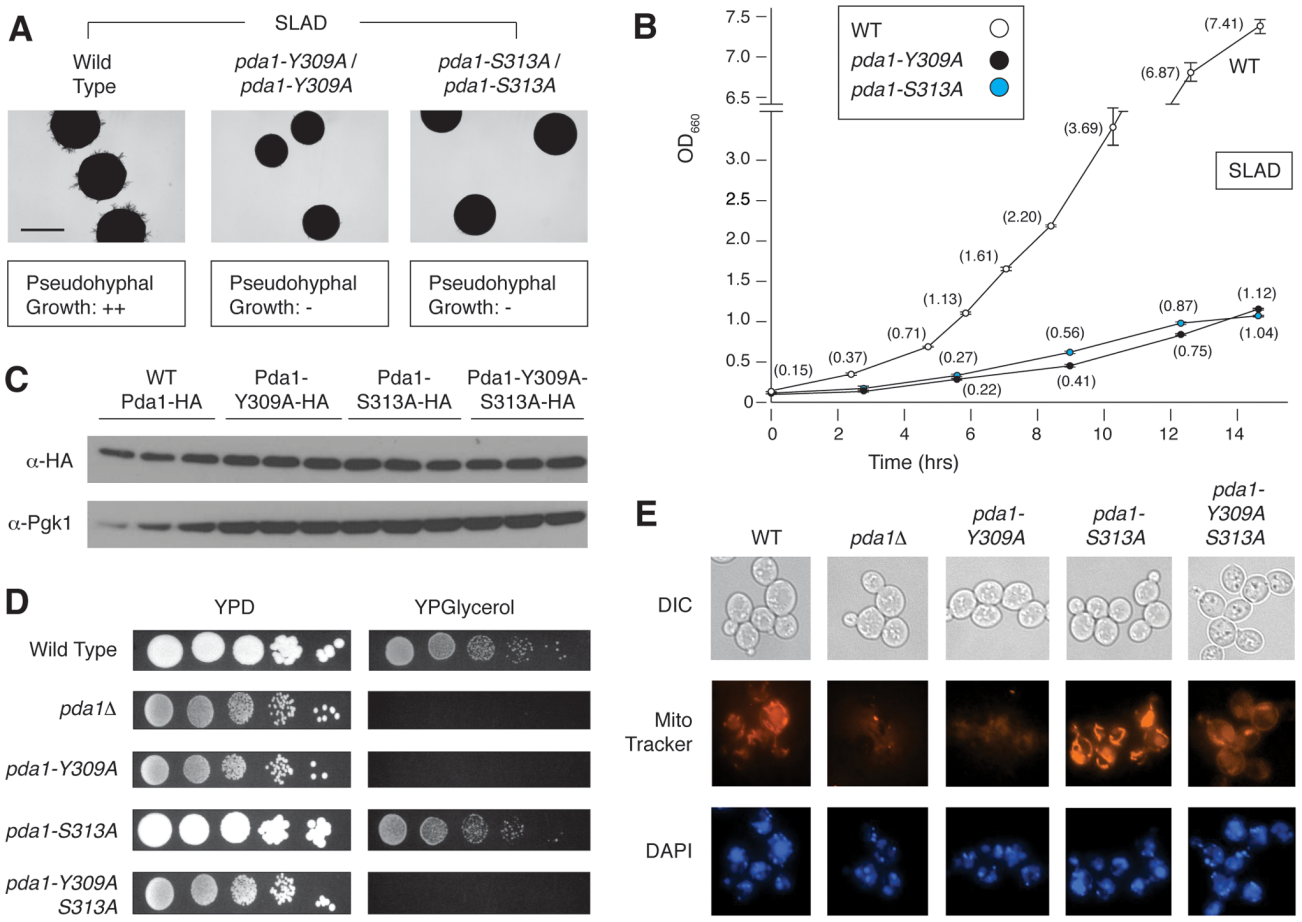
Phenotypic analysis of integrated homozygous diploid *pda1* site-directed mutants. A) The *pda1-Y309A*/*pda1-Y309A* and *pda1-S313A*/*pda1-S313A* mutants demonstrated a marked decrease in pseudohyphal differentiation on nitrogen-limited media. Scale bar, 2 mm. B) The homozygous diploid *pda1-Y309A* and *pda1-S313A* mutant strains exhibited a significant fitness defect when grown in low-nitrogen liquid media. C) Western analysis of each individual point mutant as well as the combined *pda1-Y309A-S313A* mutant indicates that Pda1p levels are comparable to those observed in a wild-type strain; thus, the observed phenotypes do not result from decreased levels of Pda1p. For this analysis, wild-type and mutant alleles of *PDA1* were HA-tagged; antibody directed against native Pgk1p was used as a control. D) Growth of the *PDA1* mutants on both fermentable (YPD) and non-fermentable media (YPGlycerol) indicates a defect in respiration in the *pda1*Δ/Δ, *pda1-Y309A*/*pda1-Y309A*, and *pda1-Y309A-S313A*/*pda1-Y309A-S313A* mutants. E) The mitochondrial content and membrane-potential-based structure of the indicated strains was imaged using DAPI and MitoTracker, respectively. Minimal changes were observed in mitochondrial content (DAPI stain, bottom row) among the strains, while specific morphological/membrane-potential defects were observed in the homozygous diploid *pda1*Δ, *pda1-Y309A*, and *pda1-Y309A-S313A* mutants (MitoTracker, middle row). Cell shape was assessed by differential interference contract (DIC) microscopy.

Of the point mutants assayed above, strains with mutations in *PDA1* exhibited pseudohyphal growth defects on low-nitrogen medium, with a much less acute growth defect in SC media. As indicated in Figure 5A and B, two distinct point mutations in *PDA1*, the *Y309A* and *S313A* alleles, yield a dramatic fitness defect and loss of pseudohyphal differentiation in nitrogen-limiting conditions. Pda1p is a subunit of the mitochondrial pyruvate dehydrogenase complex involved in the conversion of pyruvate to acetyl-CoA [54]. Cells lacking *PDA1* demonstrate diminished growth on glucose from a respiratory deficiency due to mitochondrial DNA loss [55]. We found no noticeable difference in protein levels for either single point-mutant (Y309A and S313A) or the double mutant (Y309A-S313A) relative to a wild-type strain (Figure 5C), indicating that the observed phenotypes were not due to instability of Pda1p. Interestingly, when each mutant was screened on glycerol-containing media that forced respiratory growth, the S313A mutant grew as well as wild-type, while the Y309A mutant exhibited a phenotype analogous to the *pda1*Δ/Δ mutant [55] (Figure 5D). The double Y309A-S313A mutant shared the respiratory deficiency of the Y309A mutant. We also investigated whether the mutation of these residues altered mitochondrial structure or mitochondrial DNA. By live-cell DAPI staining, no abnormal mitochondrial DNA phenotypes were observed between the wild-type strain, the *pda1*Δ/Δ mutant, or any of the site-directed mutants (Figure 5E, lower). However, staining with the membrane-potential-dependent dye MitoTracker illustrates dramatic differences in mitochondrial membrane potential or structure between these mutants and wild-type (Figure 5E, middle). The wild-type and *pda1-S313A* strains exhibit similar mitochondrial staining, while the *pda1*Δ, *pda1-Y309A*, and *pda1-Y309A-S313A* mutants all demonstrate a mitochondrial membrane phenotype. Collectively, both the Y309A and S313A mutations result in the abolishment of pseudohyphal growth in conditions of low nitrogen, and the Y309A mutation also yields phenotypes indicative of impaired respiration.

### Epistasis analysis of Sks1p with respect to glucose signaling and pseudohyphal growth

**Figure 6:**
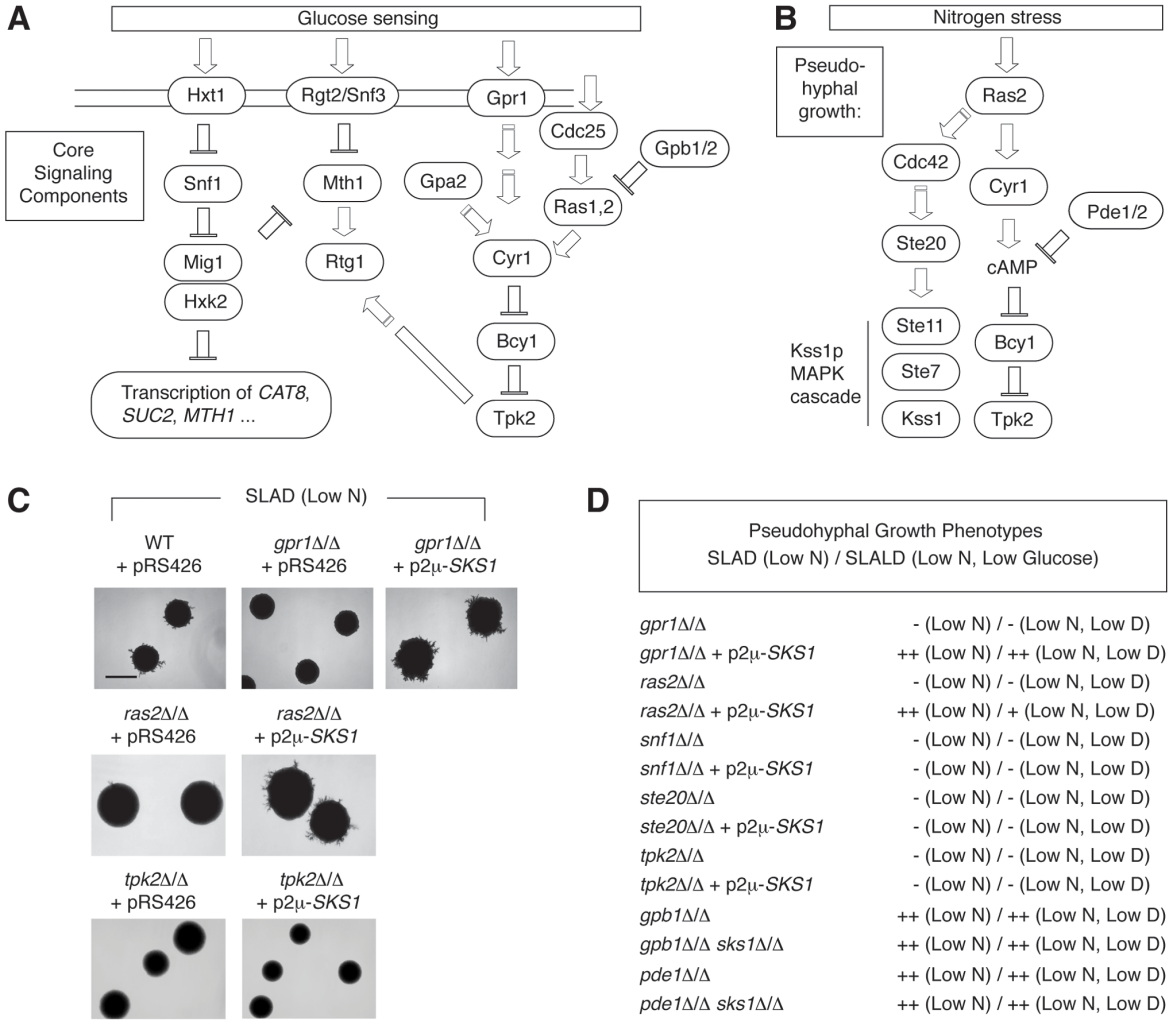
*SKS1* is epistatic with components of the nutrient-responsive cAMP-dependent/PKA pathway. A) Schematic of the core yeast glucose sensing components. B) Diagram of core signaling components of yeast pseudohyphal differentiation in response to nitrogen stress. C) Over-expression of *SKS1* from its native promoter on a 2μ plasmid restores the pseudohyphal growth phenotype of both a *gpr1*Δ/Δ and *ras2*Δ/Δ mutant strain in the Σ1278b background. Scale bar, 2 mm. D) *SKS1* overexpression does not restore pseudohyphal growth in homozygous diploid strains deleted of the PAK Ste20p upstream of the filamentous growth MAPK pathway, the PKA catalytic subunit Tpk2p, or the AMP kinase Snf1p under low nitrogen and low nitrogen/glucose conditions. The double deletion mutants *gpb1*Δ/Δ *sks1*Δ/Δ and *pde1*Δ/Δ *sks1*Δ/Δ exhibit pseudohyphal growth phenotypes that resemble *gpb1*Δ/Δ and *pde1*Δ/Δ, respectively, in SLAD and SLALD media.

The studies presented here support a role for Sks1p in enabling wild-type glucose signaling and pseudohyphal growth; however, the molecular context and genetic relationships of *SKS1* with respect to the corresponding signaling pathways is unclear. Consequently, we performed epistasis experiments examining the phenotypic consequences of over-expressing *SKS1* in diploid *S. cerevisiae* strains deficient for components of both glucose signaling pathways and pseudohyphal signaling networks (Figure 6A and B). Here, we examined whether *SKS1* could act as a high-copy suppressor of mutations in cAMP signaling (*gpr1*Δ/Δ, *ras2*Δ/Δ, and *tpk2*Δ/Δ), MAPK signaling (*ste20*Δ/Δ), or Snf1p signaling (*snf1*Δ/Δ). Each of these mutations generates a yeast strain deficient in pseudohyphal differentiation under conditions of limiting nitrogen. We found that over-expression of *SKS1* was able to suppress the *gpr1*Δ/Δ phenotype (Figure 6C). Interestingly, a *ras2*Δ/Δ mutant also demonstrated a moderate phenotypic rescue from overexpression of *SKS1*; however, *SKS1* overexpression did not restore pseudohyphal growth in a *tpk2*Δ/Δ mutant, indicating that *SKS1* acts downstream of *GPR1* and *RAS2* but upstream of or at the level of *TPK2* (Figure 6D). Overexpression of *SKS1* in the *gpr1*Δ/Δ and *ras2*Δ/Δ backgrounds did not result in filamentation on media with normal levels of nitrogen. *SKS1* did not suppress mutations in *STE20* or *SNF1*. Consistent with this *STE20* result, the *sks1*Δ/Δ mutant exhibited no loss of pseudohyphal MAPK signaling under conditions of nitrogen limitation (data not shown), as assessed using a *P_FRE(TEC1)_-lacZ* reporter system that is specifically responsive to the MAPK signaling components required for filamentous growth [26]. We also tested whether deletion of *SKS1* could affect the hyper-filamentous phenotype of strains deleted for *PDE1*, which encodes a phosphodiesterase that degrades cAMP [56], and strains deleted of *GPB1*, which encodes a Gβ-subunit of the Gpa2p heterotrimeric G protein [57]. In each case, the double deletion mutants remained hyperfilamentous, indicating no clear genetic relationship between *SKS1* and these genes.

### Mss11p and Rgt1p are required for wild-type SKS1 transcription

**Figure 7:**
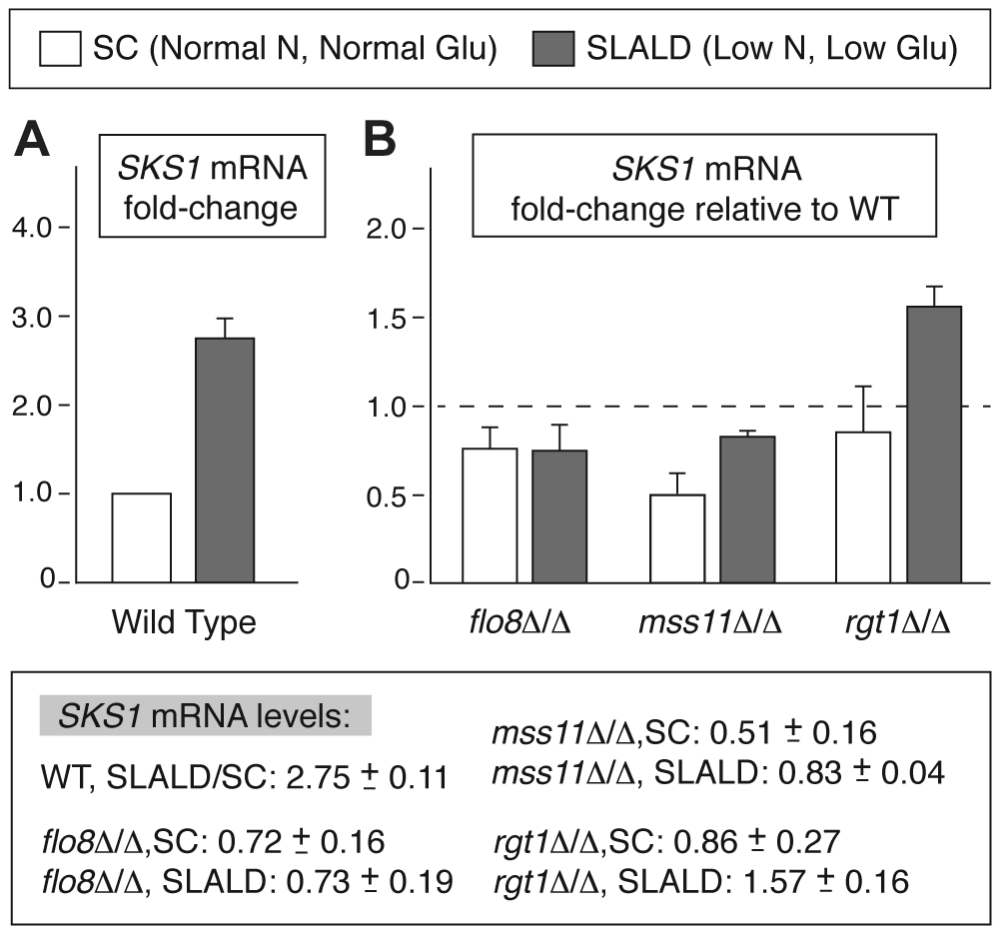
*SKS1* transcript levels are regulated in response to nitrogen and glucose levels. A) Analysis of *SKS1* mRNA levels in a wild-type strain of the filamentous Σ1278b background in normal media relative to media limited in nitrogen and glucose. B) Analysis of *SKS1* mRNA levels in the indicated homozygous diploid deletion mutants under normal and low nitrogen/glucose conditions. Fold-change in mRNA relative to wild type is indicated with standard error; actual values are listed below the graph.

In complement to our analysis of Sks1p kinase activity, we also investigated whether known transcriptional regulators of pseudohyphal development influenced the expression of *SKS1*. Analysis of *SKS1* transcription via quantitative real-time (qRT) PCR identified several results, as follows. First, *SKS1* mRNA levels were responsive to nitrogen and glucose limitation in wild-type *S. cerevisiae* of the filamentous Σ1278b background. *SKS1* transcript levels increased by nearly 180% under conditions of nitrogen limitation coupled with glucose limitation (SLALD medium) (Figure 7A). A comparison of *SKS1* transcript levels between mutants deleted for known transcriptional regulators of pseudohyphal differentiation (*flo8*Δ/Δ, *mfg1*Δ/Δ, *mga1*Δ/Δ, *mss11*Δ/Δ, *phd1*Δ/Δ, *phd1*Δ/Δ, and *tec1*Δ/Δ) and wild-type *S. cerevisiae* found that the transcription factor Mss11p, involved in glucose signaling, invasive growth, and starch degradation, as well as Flo8p, the well-known filamentous growth transcriptional activator, exhibited minor decreases in *SKS1* mRNA levels under the indicated extracellular conditions (Figure 7B). The *flo8*Δ/Δ mutant displayed an approximate 30% reduction in *SKS1* transcript levels relative to a wild-type strain in both standard and low nitrogen/glucose SLALD media. Mss11p demonstrated a reduction in *SKS1* mRNA levels of nearly 65%, but only in standard media promoting vegetative growth. We also examined the *SKS1* transcriptional response in a diploid strain deleted for *RGT1*, encoding a glucose-responsive transcriptional regulator known to repress the expression of many *HXT* genes [58], [59]. Compared to the wild-type control, the *rgt1*Δ/Δ mutant demonstrated a marked increase in *SKS1* transcript abundance under conditions of low nitrogen coupled with glucose limitation (Figure 7B).

### The *SKS1* ortholog *SHA3* is required for wild-type colony morphology in *Candida albicans*

**Figure 8:**
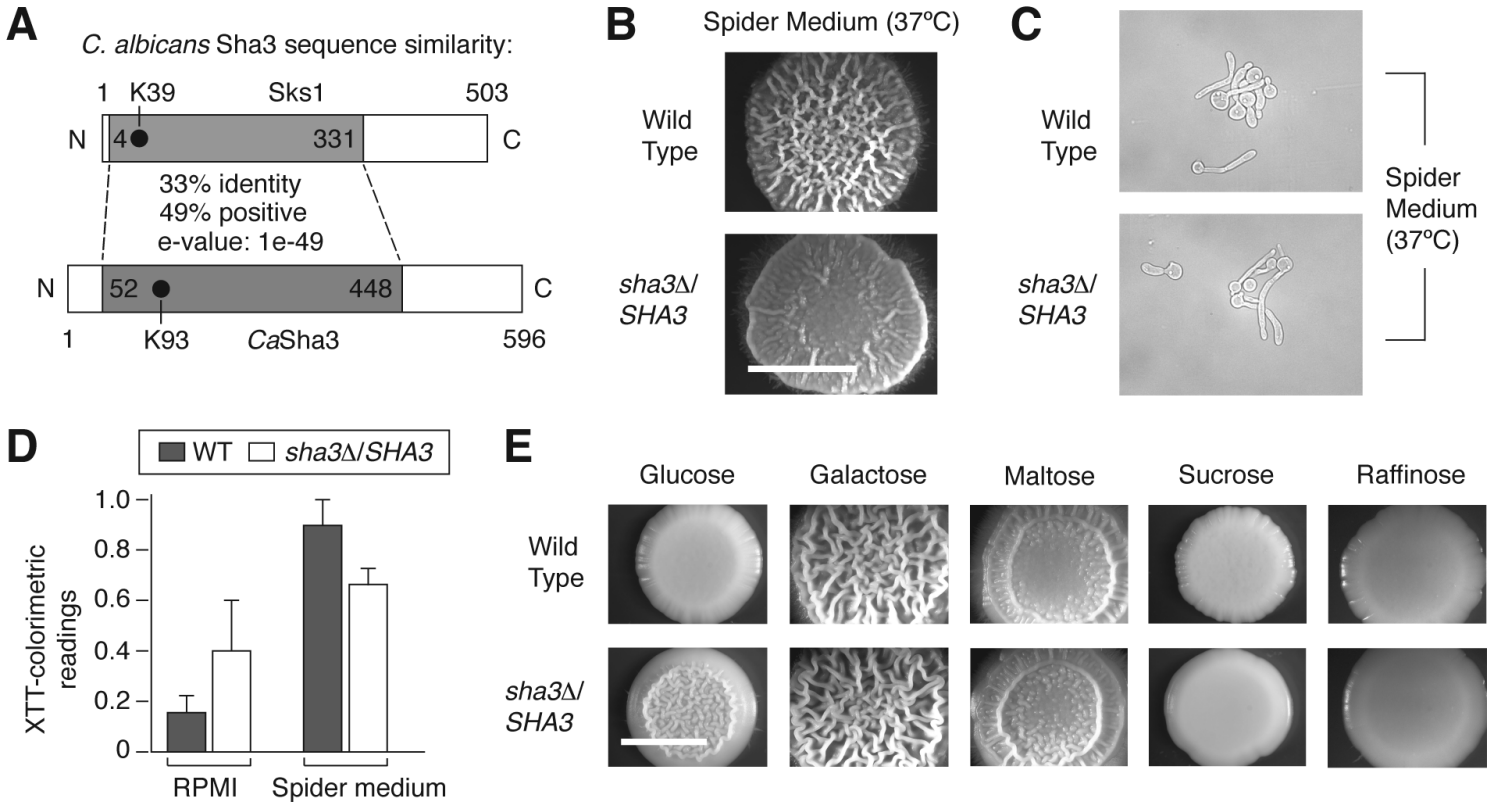
The *SKS1* ortholog *SHA3* is required for wild-type colony morphology in Spider media and in glucose-containing media in *Candida albicans*. A) Schematic diagram indicating the degree and extent of sequence similarity between Sks1p and *C. albicans* Sha3p. The shaded area is conserved, and the location of the catalytic lysine residue in each respective kinase domain is indicated. The percentage of sequence identity and similarity in the conserved shaded area is shown, along with the corresponding *e-*value associated with the alignment of these regions. B) A heterozygous deletion strain of *SHA3* exhibits decreased colony wrinkling on Spider medium relative to a wild-type strain. Spider medium contains mannitol as a carbon source. The degree of wrinkling is indicated in the inset. C) Cell morphology of wild type and *sha3*Δ/*SHA3* mutants in liquid Spider media. D) Biofilm formation in wild type and *sha3*Δ/*SHA3 C. albicans* strains in RPMI 1640 media (RPMI) and Spider media. Assays were performed on three independent biological replicates; standard error is shown. Treating growth in RPMI as a control, the ratio of biofilm formation in wild type cells in Spider media versus RPMI is 6.5 with a variance of 1.5, and for *sha3*Δ/*SHA3* mutants, the ratio is 2.1 with a variance of 0.9. E) Colony morphology of wild type and *sha3*Δ/*SHA3* strains on media containing varied carbon sources. Strains were grown in YP media supplemented with the indicated carbon source to a final concentration of 2%. Strains were grown as described for four days at 25°C prior to imaging. Scale bar, 2 mm.

*Candida albicans* is both a successful commensal and pathogen of humans, sharing with *S. cerevisiae* the ability to undergo morphological transitions in response to appropriate environmental cues [7], [60]–[62]. The importance of this morphological differentiation is underscored by the fact that hyphal development is required for virulence in *C. albicans*. The *S. cerevisiae SKS1* gene is conserved in *C. albicans*, and considering the strong conservation of pathway structure between these organisms, we hypothesized that the *SKS1* ortholog in *C. albicans* may serve a similar function in integrating environmental cues to regulate fungal morphology. To test this hypothesis, we generated a heterozygous deletion of the *SKS1* ortholog *SHA3* in the *C. albicans* strain BWP17. *SHA3* shares approximately 33% sequence identity with *SKS1* and also encodes a kinase involved in glucose transport and glucose-responsive signaling [63] (Figure 8A). On Spider growth medium in which mannitol is the carbon source, the *C. albicans SHA3* heterozygous mutant displayed a decrease in colony wrinkling relative to an isogenic wild-type strain (Figure 8B). Consistent with this result, Uhl *et al.* [64] found that a heterozygous mutant containing a transposon insertion upstream of *SHA3* in its promoter region exhibited decreased hyphal growth on Spider medium. In liquid culture, cell morphology is wild type in the *SHA3* heterozygous deletion mutant (Figure 8C), but the mutant does exhibit a statistically significant decrease in biofilm formation on Spider medium (Figure 8D). The ratio of biofilm formation on Spider media versus control RPMI buffer also indicates an approximately three-fold decrease in the *sha3*Δ*/SHA3* strain relative to wild type. Since the heterozygous *sha3* mutant exhibits a phenotype on Spider medium, we examined if colony morphology was affected on media containing other carbon sources. As indicated in Figure 8E, the *sha3*Δ*/SHA3* mutant exhibits a colony morphology distinct from wild type on medium with glucose as the carbon source and a slight phenotype on media containing sucrose.

## Discussion

Cellular adaptation to nitrogen or carbon deprivation in *S. cerevisiae* requires the remodeling of cellular metabolism and the precisely coordinated restructuring of cellular morphology. Here, we identify the glucose-responsive Sks1p kinase as a signaling protein required for pseudohyphal growth induced by nitrogen limitation and nitrogen limitation coupled with glucose limitation. Ninety-one proteins undergo Sks1p-dependent phosphorylation, and the functional scope of these phosphoproteins identifies Sks1p contribution to glucose signaling as well as additional processes and pathways required for pseudohyphal growth, including mitochondrial function. Epistasis studies indicate that *SKS1* acts downstream of *GPR1* and *RAS2*, consistent with Sks1p regulation of or by glucose-responsive cAMP signaling. *SKS1* transcript levels are dependent upon Mss11p and Rgt1p. *SKS1* is conserved, and the *SKS1* ortholog *SHA3* in *C. albicans* is required for wild-type colony morphology on glucose-containing medium and on Spider medium with mannitol as a carbon source. Collectively, these results are consistent with a function for Sks1p kinase activity in the integration of glucose-responsive signaling and filamentous development – an example of signaling crosstalk that has not been extensively studied or well understood.

In this study, we utilized a SILAC-based mass spectrometry approach to identify phosphorylation events dependent upon Sks1p kinase activity. In *S. cerevisiae*, several phosphoproteomic strategies have been utilized recently to profile differential phosphorylation [65]–[68]. In particular, Bodenmiller *et al.* [69] implemented a label-free mass-spectrometry approach to investigate the global phosphoproteomic response of *S. cerevisiae* to the systematic deletion of protein kinases and phosphatases. Trade-offs exist in considering the relative advantages of both label-free and labeling strategies. Label-free methods have been shown to identify a larger number of proteins than label-based methods; however, SILAC-based strategies typically enable better quantification and identification of differentially abundant proteins, while also providing greater reproducibility across samples [70]. It is important to bear in mind that both label-free and SILAC-based interventional phosphoproteomic methods identify direct and indirect phosphorylation events; consequently, the studies here are intended to identify the broad scope of cell processes and pathways encompassed within the Sks1p signaling network.

Notably, the study by Bodenmiller and colleagues did address the Sks1p signaling network in a non-filamentous strain under vegetative growth conditions, and approximately 30% of the proteins detected in this analysis were also identified by label-free methods in that work. Further, the overlap between the datasets is striking, in that nine proteins exhibiting Sks1p-dependent phosphorylation in a non-filamentous strain under vegetative conditions were also identified as being differentially phosphorylated in our analysis of *sks1-K39R* in a filamentous strain under conditions inducing pseudohyphal growth; these phosphoproteins include Cdc37p, Crp1p, Fyv8p, Hxt1p, Mrh1p, Mtc1p, Pda1p, Pil1p, Ptr2p, Rck2p, Zuo1p, and Ymr196w. The proteins Hxt1p, Rck2p, and Pil1p are stress-responsive, and Mrh1p, Pil1p, and Pda1p have been reported to localize to mitochondria, highlighting important processes and functions required for wild-type glucose signaling and pseudohyphal growth.

The dependence of Pda1p phosphorylation upon Sks1p and phenotypic analysis of Pda1 Y309A and S313A mutants underscores that wild-type mitochondrial membrane structure and function is interconnected with Sks1p kinase signaling. Interestingly, signaling pathways that regulate filamentation, cAMP-PKA and Snf1p, have also been shown to target mitochondria [71]–[73], and genetic screens of pseudohyphal deficient mutants have identified genes required for mitochondrial function [17], [23], [74]. The S313 residue of Pda1p is a known phosphorylation site, and phosphorylation of S313 inhibits Pda1p activity *in vitro* [75], [76]. Further, Oliveira *et al.* [77] report that the S313A mutant exhibits increased flux through pyruvate dehydrogenase during growth on glucose in a non-filamentous strain. As reported here, a filamentous strain containing the S313A mutation is able to grow on medium with glycerol as the sole carbon source. Interestingly, however, both the Pda1p S313 and Y309 residues are required for pseudohyphal growth. Sks1p is a Ser/Thr kinase; consequently, the Y309 residue in Pda1p is not expected to be a direct phosphorylation target of Sks1p. Further, Pda1p is a mitochondrial protein, and our previous analysis of Sks1p-YFP subcellular distribution did not identify mitochondrial localization [45]. Rather, we expect that Sks1p is required for wild-type phosphorylation of Pda1p Y309 because it functions in a signaling network that results in this phosphorylation event. Ongoing investigations are directed towards identifying the kinase that phosphorylates Pda1p Y309 and the mechanism by which the Y309 and S313 residues contribute to pseudohyphal growth.

Our results indicate that *SKS1* mRNA levels are glucose-regulated in the filamentous Σ1278b strain and that this regulation in SLALD medium is carried out in part by Rgt1p. Two lines of evidence support this result. First, analysis of the yeast transcriptional response to glucose by Wang *et al.* [41] indicated that *SKS1* mRNA levels increase more than two-fold when cells are switched from galactose to glucose-containing media. Second, the *snf3*Δ phenotype is subject to high copy number suppression by the *SKS1* promoter sequence, which titrates Rgt1p [78], [79]. In addition to possessing binding sites for Rgt1p, the *SKS1* promoter is reportedly bound by Mss11p and Flo8p [24], [80], although we observe that the relative individual contributions of these transcription factors to the establishment of *SKS1* mRNA levels is modest under conditions of nitrogen limitation and nitrogen/glucose limitation. Considered collectively, transcriptional regulation of *SKS1* likely results from the combinatorial contributions of numerous transcription factors. Under conditions of glucose limitation, Rgt1p actively binds target promoters to repress transcription of glucose-induced genes, and the observed increase in *SKS1* transcript levels upon *RGT1* deletion under low-glucose conditions is consistent with this observation. However, *SKS1* mRNA levels increase upon growth in SLALD media, indicating that Rgt1p cannot be predominantly responsible for the establishment of overall *SKS1* transcript levels. Additional factors, including Mss11p and Flo8p, must contribute to this transcriptional control as well, presenting a more complex picture of *SKS1* transcriptional control.

Coupling findings from this study with previous work, we suggest that Sks1p mediates cellular response to glucose limitation and nitrogen limitation by signaling through Gpr1p and the cAMP-PKA pathway. In this study, we demonstrate that *SKS1* is a high-copy suppressor of pseudohyphal-deficient *gpr1*Δ/Δ and *ras2*Δ/Δ mutants. Both Gpr1p and Ras2 are components of the cAMP-dependent PKA pathway. Gpr1p is a nutrient sensor that activates cAMP in response to low-levels of extracellular glucose [81] and regulates pseudohyphal differentiation in *S. cerevisiae* [82]. Overexpression of *SKS1* failed to restore pseudohyphal growth in a strain deleted for *TPK2*. Tk2p is one of three catalytic subunits of PKA; *TPK2* is required for pseudohyphal growth, and its function is required for the phosphorylation of Flo8p and additional key signaling events necessary for pseudohyphal differentiation [32], [83]. Thus, Sks1p may contribute to the regulation of Tpk2p or may be regulated indirectly by Tpk1p or Tpk3p. Sks1p has not been identified as a phosphoprotein, and any such mechanisms of Sks1p regulation have not been identified to date.

Pseudohyphal growth in *S. cerevisiae* is an excellent model of related processes of filamentous development in the principal opportunistic human fungal pathogen *Candida albicans*. In *C. albicans*, a variety of culture conditions, including growth on Spider medium with mannitol as a carbon source, results in the development of pseudohyphae and true hyphal tubes [9]. Orthologs of many *S. cerevisiae* pseudohyphal growth genes play similarly important roles in *C. albicans* hyphal development, and we find that the *SKS1* ortholog *SHA3* is required for wild-type colony morphology in *C. albicans* on Spider medium. The cAMP-PKA pathway is required for hyphal development and virulence in *C. albicans*, exhibiting structural similarity to the orthologous pathway in *S. cerevisiae*. Notably, *GPR1* is conserved, and Ras1p in *C. albicans* contributes to the production of cAMP through adenylate cyclase in response to various stimuli [84]. The PKA catalytic subunits Tpk1p and Tpk2p have been identified in *C. albicans*, and it will be interesting to determine if the functional relationship between *SHA3* and this cAMP-PKA pathway is similar to that which we observe in *S. cerevisiae*.

## Materials and Methods

### Yeast strains, plasmids, and growth conditions

The strains used in this study are listed in Table S1 and are isogenic derivatives of the Σ1278b strain [11], [85]. All strains were generated from the MAT**a** haploid strain Y825 (*ura3-52 leu2*Δ) and the MATα haploid strain HLY337 (*ura3-52 trp1-1*).

Standard yeast media and microbiological techniques were used [86]. Briefly, standard growth media consisted of YPD (1% yeast extract, 2% peptone, 2% glucose) or Synthetic Complete (SC) (0.67% yeast nitrogen base (YNB) without amino acids, 2% glucose, and 0.2% of the appropriate amino acid drop-out mix). Nitrogen deprivation and filamentous phenotypes were assayed using Synthetic Low Ammonium Dextrose (SLAD) medium (0.17% YNB without amino acids, 2% glucose, 50 µM ammonium sulfate and supplemented with appropriate amino acids) and Synthetic Low Ammonium Low Dextrose (SLALD) medium (0.17% YNB without amino acids, 0.05% glucose, 50 µM ammonium sulfate and supplemented with appropriate amino acids) [11], [87], [88]. Respiratory competency was assayed using YPG (1% yeast extract, 2% peptone, 3% glycerol). For plates, autoclaved 2% agar was added to the media. To promote *C. albicans* vegetative growth, 80 µg/mL of uridine was added to all media unless otherwise noted. Hyphal growth was induced in *C. albicans* via growth in Spider medium and/or 10% serum-containing medium for the indicated times at 37°C [89].

Plasmids used in this study are listed in Table S2. Plasmids pFRE-lacZ and pSKS1-K39R-vYFP were constructed as described [26], [45]. To overexpress *SKS1*, the *SKS1* open reading frame was amplified from genomic DNA and cloned into *XmaI-XhoI*-digested p426GPD [90]. The GPD promoter was then replaced with the ADH1 promoter amplified from genomic DNA (−1464 to 0) and cloned into *SacI*-*XmaI*-digested p426-GPD-*SKS1* to generate plasmid pCK020.

### Yeast gene deletions and site-directed mutagenesis

Gene deletion mutants were constructed in strains Y825 and HLY337 using a one-step PCR-based gene-disruption strategy [91], [92] with the G418 resistance cassette from plasmid pFA6a-KanMX6 [93]. Integrated point mutations were generated using the one-step site-directed mutagenesis strategy described in Zheng *et al.* [94]. After confirming the haploid mutants via PCR and site-directed mutants via sequencing, the strains were allowed to mate on YPD+G418 plates for approximately 20 hours at 30°C. Mated cells were then streaked on SC-Trp-Leu plates to select for Y825×HLY337 diploids. All yeast transformations were performed according to standard lithium acetate-mediated protocols [95]–[97].

### Surface-spread filamentation assays

Defects in surface spread filamentation were assessed as described [98]. In brief, yeast strains were grown overnight and were subsequently diluted to an OD_600_ of approximately 0.2 in fresh media. Cells were grown for at least two doublings, to an OD_600_ of approximately 0.6–1.0. Approximately 1 mL of each cell culture was transferred to a microcentrifuge tube, where the cultures were washed twice with sterile water before suspending in 1 mL sterile water and serially diluting such that the density of plating was approximately10^2^–10^3^ cells per plate; high-density plating has been shown to decrease the rate at which cells transition to the filamentous form [99]. Diluted cultures were then spread on SLAD and/or SLALD plates supplemented with appropriate amino acids and incubated at 30°C for 3 or more days. Cells were imaged using a Photometrics CoolSnapES2 digital camera mounted on a Nikon Eclipse 80i upright microscope. Colony morphology was imaged using a 4× objective, while cellular morphology was imaged with a 100× oil-immersion objective.

### Peptide sample preparation and phosphopeptide enrichment

*S. cerevisiae* Y825 control and *sks1-K39R* mutant cells were isotopically labeled with medium (Lys-4/Arg-8) amino acids during cell culture (SILAC). Cell cultures were lysed by bead beating in lysis buffer; the lysis buffer was composed of 50 mM tris buffer (pH 8.2), 8 M urea, and protease inhibitors (Roche) and phosphatase inhibitors (50 mM NaF, 50 mM beta-glycerophosphate, 1 mM sodium vanadate, 10 mM sodium pyrophosphate, 1 mM phenylmethylsulfonyl fluoride). Frozen cells were suspended in 400 µl lysis buffer and were lysed by applying three cycles of bead beating (for one minute each) with a 2-minute rest on ice between cycles. Supernatants containing protein extract were recovered by centrifugation at 14,000 g for 10 minutes, and protein concentrations were measured by Bradford assay. Equal amounts of protein from three SILAC-labeled cells were combined, treated for disulfide reduction and alkylation, and digested with TMPK-treated trypsin (Worthington Biochemical Corp., Lakewood, NJ) at a trypsin∶protein ratio of 1∶10 at 37°C overnight.

Peptide mixtures were desalted with C18 (Waters) and separated into 12 strong cation exchange (SCX) fractions on a PolySulfoethyl A column (PolyLC, 150×4 mm) over a 48 minute salt gradient with two mobile phases: 100% solvent A (5 mM KH_2_PO_4_, 30% acetonitrile, pH 2.7) for 5 minutes, a linear gradient of 0–40% solvent B (250 mM KH_2_PO_4_, 30% acetonitrile, pH 2.7) in the following 35 min, a stiff increase of 40–100% B in 3 min, and flushing with 100% B for 5 min. Collected SCX fractions were desalted with C18 (Waters) and subjected to selective phosphopeptide enrichment using ZrO_2_ (Glygen, 50 µm i.d. resin) under acidic conditions in the presence of 2,5-dihydroxy benzoic acid [100], [101]. Phosphopeptides selectively bound on ZrO_2_ were eluted with 4% NH_4_OH. The ZrO_2_ eluate of enriched phosphopeptides and the flow-through of each SCX fraction were analyzed by nanoLC-tandem mass spectrometry (MSMS).

### Mass spectrometric analysis and SILAC quantification

NanoLC-MSMS experiments were performed on a hybrid type mass spectrometer (Thermo, LTQ-Orbitrap XL) coupled to a nanoLC system (Eksigent, 2D nanoLC). Samples were separated on a custom capillary column (150 mm×75 µm, 3 µm Sepax HP-C18) using a 120 min linear aqueous gradient (9–90% acetonitrile, 0.01% formic acid) delivered at 250 nL/min. The eluent was introduced on-line to the LTQ-Orbitrap via an electrospray device (Advion, TriVersa NanoMate) in positive ion mode.

The LTQ-Orbitrap was operated in a data-dependent mode alternating a full MS scan (300–1700 m/z at 60,000 resolution power at 400 m/z) in the Orbitrap analyzer and collision-induced dissociation scans (CID-MSMS) for the 7 most abundant ions with signal intensity above 500 from the previous MS scan in LTQ. Recurring precursor ions were dynamically excluded for 30 sec by applying charge-state monitoring, ions with 1 or unassigned charge states were rejected to increase the fraction of ions producing useful fragmentation. Lock mass ([(Si(CH_3_)_2_O)_6_]^1+^, m/z = 445.120029) was used for internal calibration. Each sample was analyzed by two LC-MS experiments. Raw LC-MS data file sets were processed, database searched, and quantified using MaxQuant (ver 1.0.13.8) [102] and the Mascot search engine together. Mascot database searches were performed against a composite database of forward and reverse sequences of verified yeast open reading frames from the Saccharomyces Genome Database. Variable modifications were allowed for oxidation (M) and phosphorylations (STY), as well as a fixed modification of carbamidomethylation (C). Peptide, protein, and phosphorylation site identifications were filtered at a false discover rate of 5%. The MaxQuant normalized M/L (medium/light) ratios with significance B scores less than 0.05 were considered statistically significant. 1068 peptides were identified, corresponding to 552 distinct proteins.

The mass spectrometry proteomics data have been deposited to the ProteomeXchange Consortium (http://proteomecentral.proteomeexchange.org) via the PRIDE partner repository [103] with the dataset identifier PXD000414.

### Identification of previously unreported phosphorylation sites and network analysis

A network scaffold was constructed was constructed using interactions from the publicly available Kyoto Encyclopedia of Genes and Genomes (KEGG), GeneMania and BioGrid databases [104]–[106]. KEGG xml files for the glycolysis/gluconeogenesis (accession sce00010), cell cycle (accession sce04111), meiosis (accession sce04113) and MAPK signalling pathways (accession sce04011) were downloaded and parsed using an in-house program to create a network. The genes in the resulting network were then uploaded to GeneMania in order to retrieve additional genetic and physical interactions. Finally, the interactions for *SKS1* were downloaded from BioGrid and appended to the network.

Differentially phosphorylated proteins, identified by their differentially abundant phosphopeptides upon enrichment, were first filtered using the significance of the medium/light isotope ratios; we implemented a significance (A) cut off at or below 0.05. The resulting protein list was mapped on to the network scaffold using Cytoscape [107]. The network was clustered by node attributes to reflect the pathways from which the genes originated. As expected, the network consisted of three groups/sub-networks (glycolysis/gluconeogenesis, cell cycle/meiosis, and MAPK signaling sub-networks).

### Assays for fitness and respiratory deficiency

Yeast strains were inoculated in 5 mL SC and incubated overnight at 30°C with constant shaking (250 rpm). Cell cultures were subsequently diluted in 6 mL of SC, SLAD, and SLALD to an OD_600_ of approximately 0.1 and incubated at 30°C with constant shaking (250 rpm) for approximately 15 hours. OD_600_ measurements were collected approximately every 3 hours from the time of dilution. Full growth curve datasets for the analysis of mutants in SLAD and SLALD media are provided in Tables S3 and S4, respectively. Assays for respiratory deficiency were implemented as follows. Single colonies were inoculated in 5 mL YPD media and incubated with continuous agitation overnight. Cell cultures were diluted to an OD_600_ of approximately 0.3 in fresh YPD media and grown at 30°C with shaking for at least two doublings, to an OD_600_ of approximately 1.0. Each yeast cell culture was then adjusted to an identical OD_600_ and serially diluted 10^−1^, 10^−2^, 10^−3^, and 10^−4^, respectively. Subsequently, 5 µL of each diluted yeast culture was spotted onto YPD and YPG plates and incubated at 30°C for three to five days.

### RNA preparation and qRT-PCR analysis

Yeast strains were inoculated in 5 mL SC and incubated overnight at 30°C with constant shaking (250 rpm). Cell cultures were diluted to an OD_600_ of approximately 0.3 in fresh SC, SLAD, and SLALD media and grown at 30°C with shaking for 4 hours. Afterward, cell cultures were collected by centrifugation at 3000 g for 5 minutes; the supernatant was removed, and cell pellets were flash frozen in a dry ice/ethanol bath. Total RNA was extracted using the RiboPure Yeast Kit (Ambion) following the manufacturer's protocol. First-strand cDNA synthesis was performed using the Superscript II Reverse Transcriptase Kit (Invitrogen) with 2 µg of total RNA as template and Oligo d(T)_12–18_ as primers according to the manufacturer's protocol. Quantitative real-time assays were performed in triplicate with a Mastercycler EP Realplex4 S (Eppendorf) using SYBR Green I dye-based detection (Life Technologies). Each reaction contained 10 µL SYBR Green PCR Master Mix (Life Technologies), 0.2 µM of the appropriate primers, and 120 ng of cDNA template in a total volume of 20 µL. The real time PCR reactions were performed at 95°C for 5 minutes followed by 40 cycles of 30 seconds at 95°C, 30 seconds at 60°C, and a final step at 72°C for 30 seconds. Relative differences in RNA levels were normalized against *ACT1* levels using the delta delta C_T_ method [108].

### Western analysis

Yeast strains were analyzed by Western blotting according to standard protocols [109]. For Western analysis, 10 µL of protein sample were separated via SDS-PAGE and transferred to Immun-Blot PVDF (Bio-Rad) using standard methods. Protein detection was carried out using antibodies against Hemagglutinin (HA) (1∶2000; Abcam) in TBS+0.1% Tween20 and 5% milk. After immunodetection of Hemagglutinin, the membrane was stripped using Stripping Buffer (62.5 mM Tris pH 6.8, 100 mM β-mercaptoethanol, and 2% SDS) at 65°C for 30 minutes with occasional agitation. Normalization of loading was achieved by probing the original membrane with antibodies against yeast 3-phosphoglycerate kinase Pgk1p (1∶5000; Invitrogen) in the buffer conditions used previously.

### Observation of mitochondrial morphology

Mitochondrial morphology was scored using MitoTracker CMXR (Molecular Probes) for labeling the mitochondrial membrane and 4′,6-diamidino-2-phenylindole (DAPI) for labeling mitochondrial DNA. MitoTracker was added to 1 mL aliquots of each cell culture to a final concentration of 0.5 µM, and the samples were incubated at 30°C for 30 minutes similar to Nunnari *et al.* [110]. 3 µL of stained culture was then mixed with 3 µL of DAPI mounting media (9.25 mM p-Phenylenediamine (Sigma), 0.18 µM DAPI (Sigma), in glycerol) on a glass slide [111]–[113]. The cell suspension was then covered with a glass coverslip and imaged using a Photometrics CoolSnapES2 digital camera mounted on a Nikon Eclipse 80i upright microscope.

### Phenotypic analysis of *C. albicans sha3Δ/SHA3*

Construction of the heterozygous *C. albicans sha3::CdHIS1/SHA3* and homozygous *sha3::CdHIS1/sha3::ARG4* deletion mutants was performed using the transformation methods described in Walther *et al.* [114]. Wild-type and mutant colonies were grown overnight at 30°C in 3 ml YPD or SC media minus the appropriate amino acids and supplemented with uridine. To assess colony morphology, cell cultures were diluted to an OD_600_ of approximately 0.25 in fresh YPD+uri and grown at 30°C with shaking for at least two doublings, to an OD_600_ of approximately 0.6–1.0. 5 µl of each culture was spotted onto YPD+uri, YPD+uri+10% serum, and Spider plates. After drying on the bench, YPD+uri plates were incubated at 30°C and YPD+uri+10% serum and Spider plates were incubated at 37°C for 3–5 days.

For the study and measurement of *C. albicans* biofilm development, we used a metabolic 2,3-bis(2-methoxy-4-nitro-5-sulfophenyl)-2H-tetrazolium-5-carboxanilide (XTT) reduction assay as described in Pierce *et al.* [115] with slight modifications. Briefly, triplicate cell cultures were grown overnight at 30°C in SC media. The cultures were centrifuged, washed twice with 1× PBS and then resuspended in pre-warmed (37°C) medium (RPMI 1640-MOPS or Spider) at a final concentration of OD_520_ = 0.38, a cell concentration that was demonstrated to correlate with optimum biofilm formation [116]. Subsequently, 100 µL of each culture was added in triplicate to a 96-well plate. The plate was then incubated at 37°C with shaking (100 rpm) for 24 hours. After 24 hours, the wells were washed 3 times with 1× PBS, and100 µL of pre-warmed SC media was added to each well followed by shaking (100 rpm) at 37°C for an additional 8 hours. Post incubation, the media was removed and the XTT assay performed as described [115].

## Supporting Information

Dataset S1 Listing of phosphopeptides identified in this study by SILAC-based quantitative phosphoproteomics. Each phosphopeptide sequence is presented; “ph” indicates the predicted site of phosphorylation. The corresponding protein for each peptide is presented, with standard and systematic yeast names. The normalized ratio of M/L isotope is indicated along with the significance. Phosphopeptides identified multiple times are presented as such with their respective M/L ratios and significance measures.
(XLSX)

Dataset S2 Listing of genetic and physical interactions used to construct the Sks1p signaling connectivity network. Each binary interaction is represented in a row; the source of the interaction data (e.g., KEGG, GeneMania, BioGrid) is indicated. Standard protein names are used as available.
(XLSX)

Table S1 Strains used in this study.
(PDF)

Table S2 Plasmids used in this study.
(PDF)

Table S3 Growth curve datasets for the analysis of *S. cerevisiae* strains in low-nitrogen (SLAD) media. Cell growth is approximated by optical density readings at a wavelength of 660 nm. Optical density measurements are presented as the average of triplicate experiments.
(PDF)

Table S4 Growth curve datasets for the analysis of *S. cerevisiae* strains in low-nitrogen/low-glucose (SLALD) media. Cell growth is approximated by optical density readings at a wavelength of 660 nm. Optical density measurements are presented as the average of triplicate experiments.
(PDF)

Table S5 Growth curve datasets for the analysis of *S. cerevisiae* strains in SC media. Measurements of optical density and cell growth were as above.
(PDF)

Table S6 Average growth rates of *S. cerevisiae* strains in SC, SLAD, and SLALD media, with optical density measurements as above.
(PDF)
