# Supplementary material for: The Yeast Sks1p Kinase Signaling Network Regulates Pseudohyphal Growth and Glucose Response
Source: PLoS Genet. 2014 Mar 6;10(3):e1004183. doi: 10.1371/journal.pgen.1004183 (PMC3945295; doi:10.1371/journal.pgen.1004183)
Supplement: Table S1 — Strains used in this study. (PDF) [file pgen.1004183.s003.pdf]

Table S1. Strains used in this study

Strain	Genotype	Source
Y825	*ura3-52 leu2_0 MAT***a**	M. Snyder (Stanford, CA)
HLY337	*ura3-52 trp1-1 MAT*α	G. Fink (MIT, MA)
yCK021	*ura3-52/ura3-52 leu2_0/LEU2 TRP1/trp1-1 MAT***a***/MAT*α	This study
yCK009	*_rgt1::KanMX6/_rg11::KanMX6 ura3-52/ura3-52 leu2_0/LEU2 TRP1/trp1-1 MAT***a***/MAT*α	This study
yCK017	*_itr1::KanMX6/_itr1::KanMX6 ura3-52/ura3-52 leu2_0/LEU2 TRP1/trp1-1 MAT***a***/MAT*α	This study
yCK036	*_hxt1::KanMX6/_hxt1::KanMX6 ura3-52/ura3-52 leu2_0/LEU2 TRP1/trp1-1 MAT***a***/MAT*α	This study
yCK037	*_lrg1::KanMX6/_lrg1::KanMX6 ura3-52/ura3-52 leu2_0/LEU2 TRP1/trp1-1 MAT***a***/MAT*α	This study
yCK038	*_npr3::KanMX6/_npr3::KanMX6 ura3-52/ura3-52 leu2_0/LEU2 TRP1/trp1-1 MAT***a***/MAT*α	This study
yCK039	*_pdr5::KanMX6/_pdr5::KanMX6 ura3-52/ura3-52 leu2_0/LEU2 TRP1/trp1-1 MAT***a***/MAT*α	This study
yCK040	*_rbs1::KanMX6/_rbs1::KanMX6 ura3-52/ura3-52 leu2_0/LEU2 TRP1/trp1-1 MAT***a***/MAT*α	This study
yCK041	*_rck2::KanMX6/_rck2::KanMX6 ura3-52/ura3-52 leu2_0/LEU2 TRP1/trp1-1 MAT***a***/MAT*α	This study
yCK042	*_tpo4::KanMX6/_tpo4::KanMX6 ura3-52/ura3-52 leu2_0/LEU2 TRP1/trp1-1 MAT***a***/MAT*α	This study
yCK098	*_gpr1::KanMX6/_gpr1::KanMX6 ura3-52/ura3-52 leu2_0/LEU2 TRP1/trp1-1 MAT***a***/MAT*α	This study
yCK100	*_prb1::KanMX6/_prb1::KanMX6 ura3-52/ura3-52 leu2_0/LEU2 TRP1/trp1-1 MAT***a***/MAT*α	This study
yCK108	*_bud6::KanMX6/_bud6::KanMX6 ura3-52/ura3-52 leu2_0/LEU2 TRP1/trp1-1 MAT***a***/MAT*α	This study
yCK110	*_scp160::KanMX6/_scp160::KanMX6 ura3-52/ura3-52 leu2_0/LEU2 TRP1/trp1-1 MAT***a***/MAT*α	This study

yCK112	_sks1::KanMX6/_sks1::KanMX6 ura3-52/ura3-52 leu2_0/LEU2 TRP1/trp1-1 MATa/MATα	This study
yCK118	_ptr2::KanMX6/_ptr2::KanMX6 ura3-52/ura3-52 leu2_0/LEU2 TRP1/trp1-1 MATa/MATα	This study
yCK124	_mds3::KanMX6/_mds3::KanMX6 ura3-52/ura3-52 leu2_0/LEU2 TRP1/trp1-1 MATa/MATα	This study
yCK151	_pda1::KanMX6/_pda1::KanMX6 ura3-52/ura3-52 leu2_0/LEU2 TRP1/trp1-1 MATa/MATα	This study
yCK169	_mga1::KanMX6/_mga1::KanMX6 ura3-52/ura3-52 leu2_0/LEU2 TRP1/trp1-1 MATa/MATα	This study
yCK170	_phd1::KanMX6/_phd1::KanMX6 ura3-52/ura3-52 leu2_0/LEU2 TRP1/trp1-1 MATa/MATα	This study
yCK172	_tec1::KanMX6/_tec1::KanMX6 ura3-52/ura3-52 leu2_0/LEU2 TRP1/trp1-1 MATa/MATα	This study
yCK189	NPR3-S486A/NPR3-S486A ura3-52/ura3-52 leu2_0/LEU2 TRP1/trp1-1 MATa/MATA	This study
yCK236	_mfg1::KanMX6/_mfg1::KanMX6 ura3-52/ura3-52 leu2_0/LEU2 TRP1/trp1-1 MATa/MATA	This study
yCK237	_mss11::KanMX6/_mss11::KanMX6 ura3-52/ura3-52 leu2_0/LEU2 TRP1/trp1-1 MATa/MATA	This study
CS337	_tpk2::KanMX6/_tpk2::KanMX6 ura3-52/ura3-52 leu2_0/LEU2 TRP1/trp1-1 MATa/MATA	This study
CS343	_ras2::KanMX6/_ras2::KanMX6 ura3-52/ura3-52 leu2_0/LEU2 TRP1/trp1-1 MATa/MATA	This study
yCK263	PDA1-S313A/PDA1-S313A ura3-52/ura3-52 leu2_0/LEU2 TRP1/trp1-1 MATa/MATA	This study
yCK264	PDA1-Y309A/PDA1-Y309A ura3-52/ura3-52 leu2_0/LEU2 TRP1/trp1-1 MATa/MATA	This study
yCK274	ITR1-S26A/ITR1-S26A ura3-52/ura3-52 leu2_0/LEU2 TRP1/trp1-1 MATa/MATA	This study
yCK275	SKS1-K39R/SKS1-K39R ura3-52/ura3-52 leu2_0/LEU2 TRP1/trp1-1 MATa/MATA	This study
yCK283	LRG1-S605A/LRG1-S605A ura3-52/ura3-52	This study

	*leu2_0/LEU2 TRP1/trp1-1 MAT***a***/MATA*	
yCK284	*PDA1-Y309A-S313A/PDA1-Y309A-S313A ura3-52/ura3-52 leu2_0/LEU2 TRP1/trp1-1 MAT***a***/MATA*	This study
yCK293	*BUD6-S347A/BUD6-S347A ura3-52/ura3-52 leu2_0/LEU2 TRP1/trp1-1 MAT***a***/MATA*	This study
yCK300	*PDA1-3HA-KanMX6 ura3-52 leu2_0 MAT***a**	This study
yCK302	*PDA1-Y309A-3HA-KanMX6 ura3-52 leu2_0 MAT***a**	This study
yCK304	*PDA1-S313A-3HA-KanMX6 ura3-52 leu2_0 MAT***a**	This study
yCK306	*PDA1-Y309A-S313A-3HA-KanMX6 ura3-52 leu2_0 MAT***a**	This study
BWP17	*ura3::_imm434/ura3::_imm434 his1::hisG/his1::hisG arg4::hisG/arg4::hisG*	FGSC (UMKC, MO)
yCK307	*sha3::CdHIS1/SHA3 ura3::_imm434/ura3::_imm434 his1::hisG/his1::hisG arg4::hisG/arg4::hisG*	This Study
