# Supplementary material for: The Yeast Sks1p Kinase Signaling Network Regulates Pseudohyphal Growth and Glucose Response
Source: PLoS Genet. 2014 Mar 6;10(3):e1004183. doi: 10.1371/journal.pgen.1004183 (PMC3945295; doi:10.1371/journal.pgen.1004183)
Supplement: Table S2 — Plasmids used in this study. (PDF) [file pgen.1004183.s004.pdf]

Table S2. Plasmids used in this study

Plasmid	Description	Source
pFRE-LacZ	$P_{FRE(TEC1)}$::*lacZ*, *URA3*, 2μ, Amp^r	Madhani and Fink, 1997
pSKS1-vYFP	P_{SKS1}-*SKS1*, *URA3*, Cen, Amp^r	Bharuca *et al*, 2008
pSKS1-KD-vYFP	P_{SKS1}-*SKS1-K39R*, *URA3*, Cen, Amp^r	Bharuca *et al*, 2008
p426GPD	P_{GPD3}, *URA3*, 2μ, Amp^r	Mumberg *et al*, 1995
pCK020	P_{ADH1}-*SKS1*, *URA3*, 2μ, Amp^r	This study
