# Supplementary material for: The Yeast Sks1p Kinase Signaling Network Regulates Pseudohyphal Growth and Glucose Response
Source: PLoS Genet. 2014 Mar 6;10(3):e1004183. doi: 10.1371/journal.pgen.1004183 (PMC3945295; doi:10.1371/journal.pgen.1004183)
Supplement: Table S3 — Growth curve datasets for the analysis of S. cerevisiae strains in low-nitrogen (SLAD) media. Cell growth is approximated by optical density readings at a wavelength of 660 nm. Optical density measurements are presented as the average of triplicate experiments. (PDF) [file pgen.1004183.s005.pdf]

Table S3. Growth curve of *S. cerevisiae* strains in SLAD media

Yeast strain	OD_{660} per time point					
	0 hr	2.5 hr	5.5 hr	8.75 hr	12 hr	14.5 hr
Wild-type	0.149	0.373	1.130	2.199	6.870	7.410
*bud6*Δ/Δ	0.085	0.199	0.646	1.643	3.100	3.830
*hxt1*Δ/Δ	0.124	0.207	0.570	1.238	2.650	3.910
*itr1*Δ/Δ	0.143	0.183	0.429	0.879	1.370	2.490
*lrg1*Δ/Δ	0.171	0.237	0.544	1.038	3.210	3.140
*mds3*Δ/Δ	0.134	0.202	0.567	1.331	2.400	3.530
*npr3*Δ/Δ	0.120	0.171	0.312	0.443	1.370	2.900
*pda1*Δ/Δ	0.127	0.167	0.210	0.214	0.391	0.485
*pdr5*Δ/Δ	0.134	0.167	0.397	0.606	0.736	1.069
*prb1*Δ/Δ	0.109	0.177	0.314	0.531	0.728	0.841
*ptr2*Δ/Δ	0.114	0.201	0.354	0.586	0.769	1.085
*rbs1*Δ/Δ	0.111	0.241	0.450	0.690	0.887	1.204
*rck2*Δ/Δ	0.101	0.265	0.522	0.886	1.688	1.478
*scp160*Δ/Δ	0.101	0.193	0.435	0.587	0.880	1.140
*tpo4*Δ/Δ	0.116	0.190	0.395	0.523	0.780	1.006
bud6-S347A	0.130	0.160	0.379	0.652	0.979	1.178
itr1-S26A	0.102	0.170	0.488	0.827	1.301	1.750
lrg1-S605A	0.118	0.199	0.413	0.688	1.027	1.300
npr3-S486A	0.109	0.176	0.378	0.676	0.990	1.038
pda1-Y309A	0.104	0.121	0.221	0.411	0.747	1.115
pda1-S313A	0.130	0.142	0.269	0.563	0.871	1.043
pda1-Y309A-S313A	0.099	0.158	0.335	0.611	1.026	1.385
