# Supplementary material for: The Yeast Sks1p Kinase Signaling Network Regulates Pseudohyphal Growth and Glucose Response
Source: PLoS Genet. 2014 Mar 6;10(3):e1004183. doi: 10.1371/journal.pgen.1004183 (PMC3945295; doi:10.1371/journal.pgen.1004183)
Supplement: Table S4 — Growth curve datasets for the analysis of S. cerevisiae strains in low-nitrogen/low-glucose (SLALD) media. Cell growth is approximated by optical density readings at a wavelength of 660 nm. Optical density measurements are presented as the average of triplicate experiments. (PDF) [file pgen.1004183.s006.pdf]

Table S4. Growth curve of *S. cerevisiae* strains in SLALD media

Yeast strain	OD_{660} per time point					
	0 hr	2.5 hr	5.5 hr	8.75 hr	12 hr	14.5 hr
Wild-type	0.138	0.348	0.985	1.340	4.000	3.340
*bud6*Δ/Δ	0.143	0.155	0.552	1.185	2.090	2.520
*hxt1*Δ/Δ	0.120	0.232	0.559	0.951	1.730	1.730
*itr1*Δ/Δ	0.109	0.208	0.438	0.645	1.486	1.970
*lrg1*Δ/Δ	0.124	0.173	0.447	0.710	1.071	1.200
*mds3*Δ/Δ	0.101	0.206	0.542	0.888	1.853	2.610
*npr3*Δ/Δ	0.135	0.159	0.284	0.408	0.734	1.100
*pda1*Δ/Δ	0.097	0.136	0.185	0.236	0.294	0.346
*pdr5*Δ/Δ	0.093	0.198	0.383	0.560	0.620	0.894
*prb1*Δ/Δ	0.107	0.203	0.397	0.569	0.687	0.800
*ptr2*Δ/Δ	0.095	0.158	0.382	0.554	0.699	0.767
*rbs1*Δ/Δ	0.075	0.215	0.459	0.595	0.719	0.984
*rck2*Δ/Δ	0.098	0.250	0.461	0.655	0.800	1.012
*scp160*Δ/Δ	0.105	0.216	0.429	0.528	0.700	0.853
*tpo4*Δ/Δ	0.112	0.173	0.356	0.539	0.663	0.870
bud6-S347A	0.130	0.137	0.350	0.540	0.747	0.835
itr1-S26A	0.117	0.207	0.375	0.631	0.888	1.010
lrg1-S605A	0.093	0.189	0.310	0.520	0.740	0.850
npr3-S486A	0.128	0.139	0.284	0.460	0.629	0.720
pda1-Y309A	0.107	0.117	0.178	0.256	0.302	0.352
pda1-S313A	0.106	0.161	0.252	0.431	0.607	0.740
pda1-Y309A-S313A	0.103	0.132	0.313	0.440	0.620	0.725
