# Supplementary material for: The Yeast Sks1p Kinase Signaling Network Regulates Pseudohyphal Growth and Glucose Response
Source: PLoS Genet. 2014 Mar 6;10(3):e1004183. doi: 10.1371/journal.pgen.1004183 (PMC3945295; doi:10.1371/journal.pgen.1004183)
Supplement: Table S5 — Growth curve datasets for the analysis of S. cerevisiae strains in SC media. Measurements of optical density and cell growth were as above. (PDF) [file pgen.1004183.s007.pdf]

Table S5. Growth curve of *S. cerevisiae* strains in SC media

Yeast strain	OD_{660} per time point					
	0 hr	2.5 hr	5.5 hr	8.75 hr	12 hr	14.5 hr
Wild-type	0.183	0.366	1.365	3.015	10.050	13.680
*bud6*Δ/Δ	0.061	0.225	0.725	2.265	6.450	11.025
*hxt1*Δ/Δ	0.138	0.235	0.742	2.195	13.100	21.255
*itr1*Δ/Δ	0.137	0.223	0.569	1.617	8.260	14.925
*lrg1*Δ/Δ	0.111	0.189	0.660	1.709	12.770	21.450
*mds3*Δ/Δ	0.129	0.236	0.659	2.093	11.710	16.200
*npr3*Δ/Δ	0.122	0.233	0.639	1.842	12.090	19.350
*pda1*Δ/Δ	0.109	0.123	0.301	0.833	2.425	5.120
*pdr5*Δ/Δ	0.087	0.156	0.358	1.602	4.105	9.440
*prb1*Δ/Δ	0.113	0.151	0.378	1.526	4.030	9.140
*ptr2*Δ/Δ	0.111	0.181	0.436	1.406	4.200	9.490
*rbs1*Δ/Δ	0.117	0.223	0.400	1.740	4.425	9.950
*rck2*Δ/Δ	0.092	0.247	0.549	2.060	5.335	9.640
*scp160*Δ/Δ	0.106	0.175	0.453	1.492	4.150	8.790
*tpo4*Δ/Δ	0.098	0.160	0.364	1.520	5.340	11.280
bud6-S347A	0.120	0.213	0.542	1.729	6.960	10.440
itr1-S26A	0.110	0.266	0.637	1.961	6.150	11.950
lrg1-S605A	0.128	0.215	0.530	1.690	5.855	12.940
npr3-S486A	0.103	0.197	0.498	1.640	5.230	15.600
pda1-Y309A	0.102	0.146	0.364	1.003	2.820	7.710
pda1-S313A	0.112	0.194	0.459	1.528	4.840	11.820
pda1-Y309A-S313A	0.104	0.204	0.537	1.694	5.400	14.400
