# Supplementary material for: The Yeast Sks1p Kinase Signaling Network Regulates Pseudohyphal Growth and Glucose Response
Source: PLoS Genet. 2014 Mar 6;10(3):e1004183. doi: 10.1371/journal.pgen.1004183 (PMC3945295; doi:10.1371/journal.pgen.1004183)
Supplement: Table S6 — Average growth rates of S. cerevisiae strains in SC, SLAD, and SLALD media, with optical density measurements as above. (PDF) [file pgen.1004183.s008.pdf]

Table S6. Average growth rates of *S. cerevisiae* strains in SC, SLAD, and SLALD media

	SC media (OD_{660}/hr)	SLAD media (OD_{660}/hr)	SLALD media (OD_{660}/hr)
Wild-type	0.94	0.51	0.23
*bud6*Δ/Δ	0.76	0.26	0.17
*hxt1*Δ/Δ	1.47	0.27	0.12
*itr1*Δ/Δ	1.03	0.17	0.14
*lrg1*Δ/Δ	1.48	0.22	0.09
*mds3*Δ/Δ	1.12	0.24	0.18
*npr3*Δ/Δ	1.33	0.20	0.08
*pda1*Δ/Δ	0.35	0.03	0.02
*pdr5*Δ/Δ	0.65	0.07	0.06
*prb1*Δ/Δ	0.63	0.06	0.06
*ptr2*Δ/Δ	0.65	0.07	0.05
*rbs1*Δ/Δ	0.69	0.08	0.07
*rck2*Δ/Δ	0.66	0.10	0.07
*scp160*Δ/Δ	0.61	0.08	0.06
*tpo4*Δ/Δ	0.78	0.07	0.06
bud6-S347A	0.72	0.08	0.06
itr1-S26A	0.82	0.12	0.07
lrg1-S605A	0.89	0.09	0.06
npr3-S486A	1.08	0.07	0.05
pda1-Y309A	0.53	0.08	0.02
pda1-S313A	0.82	0.07	0.05
pda1-Y309A-S313A	0.99	0.10	0.05
